# Effect of auriculotherapy on chronic low back pain: A protocol for systematic review and meta-analysis

**DOI:** 10.1097/MD.0000000000019722

**Published:** 2020-04-03

**Authors:** Guilong Zhang, Leixiao Zhang, Yanli Deng, Yuquan Shen, Xinling Wang, Yang Yu

**Affiliations:** a Affiliated Hospital of Chengdu University of Traditional Chinese Medicine; b Acupuncture and Tuina School, Chengdu University of Traditional Chinese Medicine; c Sichuan Second Chinese Medicine Hospital; d The First People's Hospital of Longquanyi District, Chengdu, Sichuan, China.

**Keywords:** auriculotherapy, chronic low back pain, protocol, systematic review

## Abstract

**Background:**

Chronic low back pain (CLBP) is a clinically common and recurrent disease. However, many trials have shown that auriculotherapy (AT) can effectively treat CLBP. There are currently no systematic reviews of this therapy. The plan aims to evaluate the effectiveness and safety of this treatment in patients with CLBP.

**Methods:**

This systematic evaluation will entail an electronic and manual search of all AT for CLBP from inception to January 31, 2020, regardless of the publication status or language. Databases include PubMed, Excerpt Medica Database, Springer, Web of Science, the Cochrane Library, the World Health Organization International Clinical Trial Registration Platform, the Chinese Medicine Database, the China National Knowledge Infrastructure, the Chinese Biomedical Literature Database, the China Science Journal Database, and the Wanfang Database. Other sources of information, including bibliographies and meeting minutes for identified publications, will also be searched. A manual search for grey literature, including unpublished conference articles will be performed. Additionally, any clinical randomized controlled trials related to AT for CLBP, regardless of the publication status and language limitations, will be included in the study. Study selection, data extraction, and research quality assessments will be conducted independently by 2 researchers. The main result was the use of a visual analog scale, a short pain scale, or other effective scale. Secondary outcomes included effectiveness, Oswestry dysfunction index, self-rating anxiety scale, self-depression rating scale, Pittsburgh sleep quality index, follow-up relapse rate, and adverse events. The system searches for randomized controlled trials of this therapy for CLBP. Implement the Cochrane RevMan V5.3 bias assessment tool to assess bias risk, data integration risk, meta-analysis risk, and subgroup analysis risk (if conditions are met). Mean difference, standard mean deviation, and binary data will be used to represent continuous results.

**Results:**

This study will provide a comprehensive review and evaluation of the available evidence for the treatment of CLBP using this therapy.

**Conclusion:**

This study will provide new evidence to evaluate the effectiveness and side effects of AT on CLBP. Because the data is not personalized, no formal ethical approval is required.

**PROSPERO registration number:**

CRD42020151584.

## 1 Introduction

### 1.1 Description of the condition

Low back pain (LBP) is defined as pain and discomfort, localized below the costal margin and above the inferior gluteal folds, with or without leg pain. Chronic low back pain (CLBP) as LBP persisting for 12 weeks or more.^[1–3]^ It is a major health problem leading to more years lived with disability than any other musculoskeletal condition.^[4,5]^

The lifetime prevalence of CLBP is reported as over 70% in industrialized countries (1-year prevalence 15%–45%, adult incidence 5% per year). Peak prevalence occurs between ages 35 and 55. Symptoms, pathology, and radiological appearances are poorly correlated. Pain is not attributable to pathology or neurological encroachment in about 85% of people. About 4% of people seen with CLBP in primary care have compression fractures and about 1% has a neoplasm. Ankylosing spondylitis and spinal infections are rarer. The prevalence of prolapsed intervertebral disc is about 1% to 3%.^[6]^ Risk factors are poorly understood. The most frequently reported are heavy physical work, frequent bending, twisting, lifting, pulling and pushing, repetitive work, static postures, and vibrations. Psychosocial risk factors include stress, distress, anxiety, depression, cognitive dysfunction, pain behavior, job dissatisfaction, and mental stress at work.^[7,8]^

The societal and economic costs of CLBP are high, and indirect costs are usually higher than direct medical costs.^[9–11]^ In Australia, the total cost for CLBP was estimated at AUD$9 billion in 2001, but only 11% of this amount was accounted for by direct health care costs.^[12,13]^ Similar proportions have been observed in the Netherlands and the United Kingdom.^[14,15]^ Although the costs associated with CLBP in the Netherlands have reduced from €4.3 billion in 2002 to €3.5 billion in 2007, the costs are still substantial and constituted 0.6% of the gross national product in 2007. In all these estimates, the majority of costs were attributed to productivity losses. In this regard, the Institute of Medicine reports that CLBP-related costs in USA amount to about 34 billion dollars per year.^[16]^

Auriculotherapy (AT), an adjunct to acupuncture, is based on the same ancient Traditional Chinese Medicine (TCM) as acupuncture and uses acupoints on specific areas of the inner and outer ear lobe to treat disease/illness. In TCM, a disease is considered to be caused by the imbalance of a person's energy, Qi. The stimulation of auricular acupoints regulates Qi, activates the meridians and collateral systems, and has been successful in treating health problems.^[17]^ A growing number of studies have confirmed the efficacy of AT in the treatment of CLBP.^[18,19]^ But there has been no systematic review of the treatment. Therefore, the purpose of this systematic review evaluation is to evaluate the efficacy and safety of AT for CLBP.

### 1.2 Description of the intervention

In the 1950s, a French neurosurgeon, Dr Paul Nogier, theorized that the ear represents the inverted fetus within the womb, and proposed the somatotopic correspondence of specific parts of the body to specific parts of the ear, the current AT practiced worldwide is based on Nogier theory.^[17,20]^ The World Health Organization considers auricular medicine a form of micro acupuncture that can affect the whole body.^[21]^ AT, also called auricular acupoint pressure or ear stimulation, is a method of diagnosing and treating diseases by stimulation of specific acupoints on the external ear, includes electrical stimulation, acupoint acupressure, different type of needles, seeds, and magnetic balls.^[22]^ Auricular point acupressure is a proven AT, which utilizes very tiny botanical plant seeds (eg, approximately 2 mm size) taped onto the patient s ear for acupoint stimulation. Once applied by a qualified therapist, the taped seeds remain in place for up to 1 to 3 weeks, depending on the subjects skin condition. The patient is instructed to apply pressure to the taped seed when experiencing pain.^[23]^

### 1.3 How the intervention might work?

According to TCM, CLBP is caused by external trauma, or by internal deficiency of antipathogenic Qi and external invasion by wind-cold or cold-damp, resulting in obstruction of Qi and blood in the meridians. Treatment principles include the promotion of circulation of Qi and blood, relieving pain, relaxing the muscles, and activating the blood circulation in the collaterals. The human ear plays an important role in the traditional treatment. It is widely believed that the ear is related to all parts of the human body as well as internal organs, and that stimulating the auricular acupoints can activate meridians and collaterals thus making this method suitable for treating many disorders of the body. AT, being one of the approaches in TCM, is a therapeutic method by which specific points on the auricle are stimulated to treat various disorders.^[24]^ AT is based on reflex theory, which posits that the symptomatic body can induce tenderness in specific ear points, and the treatment of these ear points can stimulate the brain to correct related pathological reflex centers, which, in turn, will induce reflex reactions in the body to relieve pathology.^[25]^

### 1.4 Objectives

To develop treatment recommendations, we systematically evaluated the efficacy and safety of AT for CLBP.

## 2 Methods

### 2.1 Study registration

PROSPERO registration number is CRD42020151584. This protocol report is structured according to the preferred reporting items for systematic reviews and meta-analyses protocols (PRISMA-P) statement guidelines.^[26]^ The review will be conducted in accordance with the PRISMA guidelines and follows the recommendations of the Cochrane Handbook for Systematic Reviews of Interventions.^[27,28]^ If we refine the procedures described in this protocol, we will update the record in the PROSPERO and disclose them in future publications related to this study.

### 2.2 Inclusion criteria for study selection

#### 2.2.1 Types of study

To evaluate the efficacy of AT in the treatment of CLBP, this paper only reviewed the randomized controlled trial (RCT) between AT and the control group. In addition, both Chinese and English publications are subject to language restrictions. All RCT that are not subject to publication state constraints will be included. If the experiment shows that the phrase is random and the blind method is not restricted, it will be regarded as a random study. Animal mechanism studies, case reports, self-controlled, non-RCTs, random crossover studies, and quasi-randomized trials will be excluded.

#### 2.2.2 Types of participants

Regardless of gender, age, ethnicity, education, and economic status, patients with CLBP who meet the following diagnostic criteria (eg, noninvasive treatments for acute, subacute, and CLBP: A Clinical Practice Guideline From the American College of Physicians) will be included.^[3]^

#### 2.2.3 Types of intervention

The experimental group should be treated with AT including auricular acupuncture or auricular acupressure or auricular acupuncture with electrical stimulation, and acupoints used according to TCM nomenclature. The types of seed used and the duration of treatment will be unlimited. AT combined with other conventional therapy should be excluded.

The following treatment comparisons will be investigated:

(1) AT compared with no treatment;
(2) AT compared with placebo or sham treatment;
(3) AT compared with other active therapies;
(4) AT in addition to active therapy compared with the same active therapy.

We will assess and compare the AT according to how the acupuncturists have been trained and educated, on their clinical experience, on total numbers of AT sessions, and on the treatment duration and frequency, and so on.

#### 2.2.4 Types of outcome measures

The primary outcome was the visual analog scale, the brief pain inventory-short form, or other validated scales used to improve CLBP after at least 2 weeks of treatment.^[29,30]^ Secondary outcomes include Response rate, Oswestry disability index, self-rating anxiety scale, self-rating depression scale, Pittsburgh sleep quality index, recurrence rate during the follow-up period, and adverse events.^[18,31,32]^ The system review will be performed independently.

### 2.3 Data sources

Our systematic review will search all RCTs for AT on CLBP, electronically and manually, regardless of publication status and language, by January 31, 2020. Databases include: PubMed, Excerpt Medica Database, Springer, Web of Science, Cochrane Library, WHO International Clinical Trials Registry Platform, Traditional Chinese Medicine databases, China National Knowledge Infrastructure, China Biomedical Literature Database, Chinese Scientific Journal Database, and Wan-Fang database. Other sources, including reference lists of identified publications and meeting minutes, will also be searched. Manually search for grey literature, including unpublished conference articles.

### 2.4 Search strategy

The search strategy will be followed the PRISMA guidelines. The key search terms are (“chronic low back pain” OR “back pain” OR “lumbar pain”) AND (“auricular acupuncture” OR “auricular acupressure” OR “auricular pressing” OR “auricular needle” OR “auricular plaster”) AND (“randomized”). The search strategy will be adapted to different databases demands. Search strategy in PubMed is shown in Table 1.

**Table 1:**
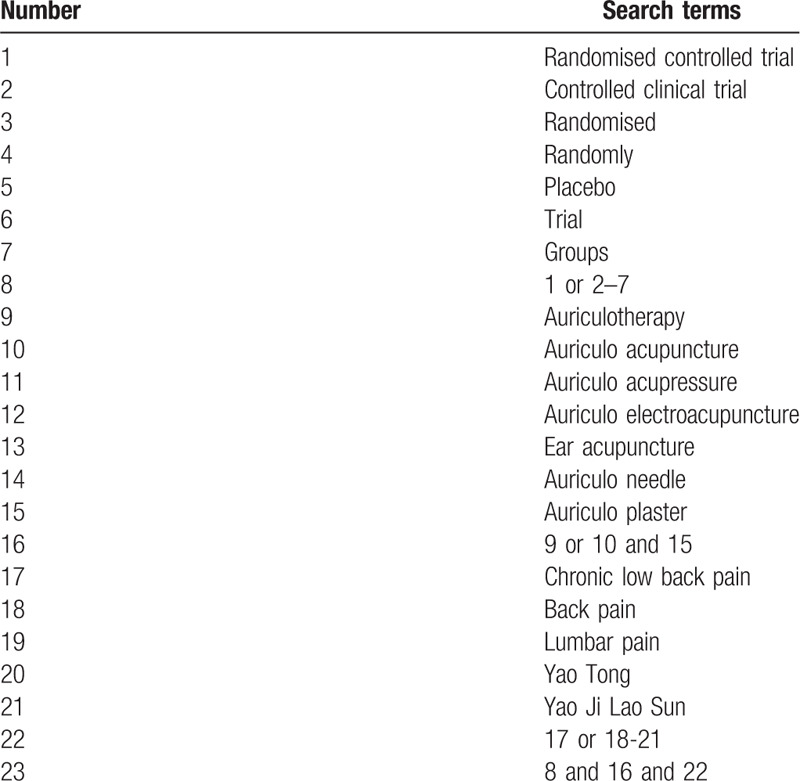
PubMed search strategy.

### 2.5 Data collection and analysis

#### 2.5.1 Selection of studies

**Figure 1:**
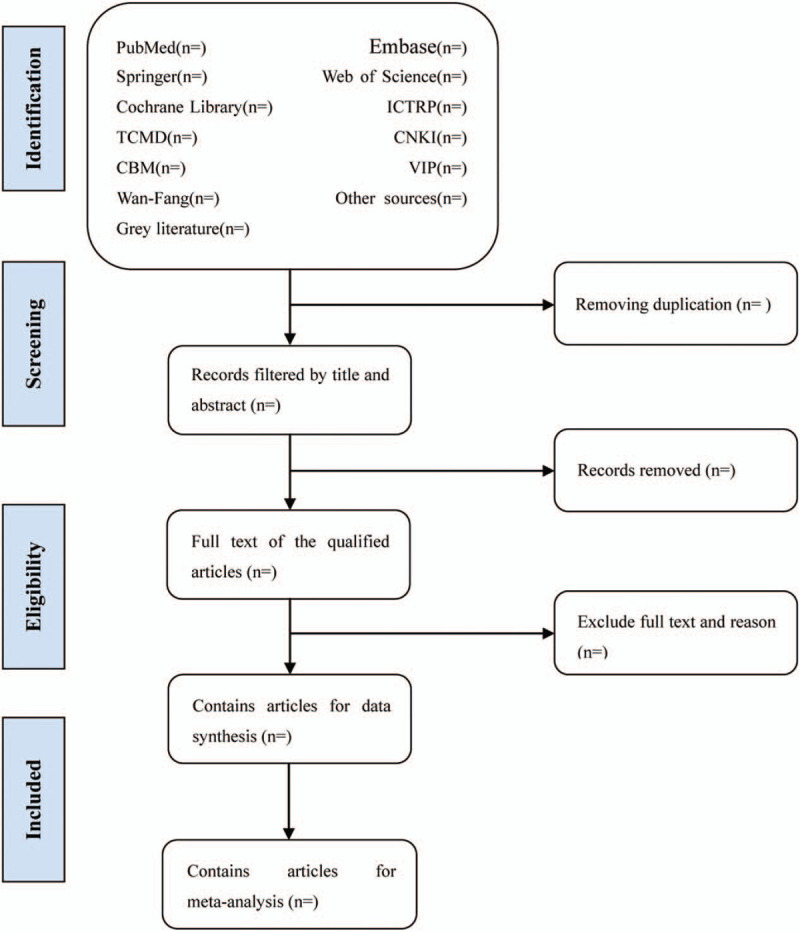
Flow diagram of studies identified.

Before literature retrieval, all reviewers are trained to ensure a basic understanding of the background and purpose of the review. In the literature screening process, we will use EndNote software (V.X8) document management software. The 2 comment author (GLZ and LXZ) will be in strict accordance with the inclusion criteria, independent screen all retrieval research, read the title, abstract and keywords in the literature, and determine which meet the inclusion criteria. We will obtain the full text of all relevant studies for further evaluation. Excluded studies will be documented and explained. If there is a disagreement in the selection process, it will be discussed by the 2 authors (GLZ and LXZ) and the third author (YY) and arbitrated if necessary. If necessary, we will contact the trial author for clarification. The primary selection process is shown in a PRISMA flow chart (Fig. 1).

#### 2.5.2 Data extraction and management

The authors will extract data independently from the selected report or study and fill out the data extraction form. We will obtain data on general information, participants, methods, interventions, outcomes, results, adverse events, conflicts of interest, ethical recognition, and other information. For publications with insufficient or ambiguous data, we will attempt to obtain information from the corresponding authors by e-mail or telephone. Any differences will be resolved through discussions between the 2 authors, and further differences will be arbitrated by the third author (YY).

#### 2.5.3 Assessment of risk of bias and reporting of study quality

The authors (YLD and YQS) will use the Cochrane Collaboration's bias risk assessment tool to assess the risk of bias in all included studies. We will assess the risk of bias in the following areas: sequence generation, assignment sequence hiding, the blindness of participants and staff, and result evaluators, incomplete outcome data, selective outcome reporting, and other sources of bias. This review uses L, U, and H as the key to these assessments, where L (low) indicates a lower risk of bias, U (unclear) indicates that the risk of bias is uncertain, and H (high) indicates a higher risk of bias. If inconsistent results appear, the final decisions will be made by the third author (YY). Information on the risk of biased assessments included in the study is summarized in tabular form and the results and impacts are critically discussed. If the information is ambiguous, we will try to contact the author. For repeated publications, we only select the original text.

#### 2.5.4 Measures of treatment effect

Data analysis and quantitative data synthesis will be performed using RevMan V.5.3. For continuous data, if there is no heterogeneity, we will use mean difference or standard mean difference to measure the therapeutic effect of 95% confidence interval (CI). If significant heterogeneity is found, a random effects model will be used. For dichotomous data, we will use the 95% CI risk ratio for analysis.

#### 2.5.5 Unit of analysis issues

We will include data from parallel group design studies for meta-analysis. Only the first phase of the data will be included in the random crossover trial. In these trials, participants were randomly divided into 2 intervention groups and individual measurements for each outcome of each participant were collected and analyzed.

#### 2.5.6 Management of missing data

If the primary result has missing or incomplete data, we will contact the author of the communication to obtain the missing data. If it is never available, exclude the experiment from the analysis.

#### 2.5.7 Assessment of heterogeneity

We will use the Review Manager to assess efficacy and publication bias (version 5.3, Nordic Cochrane Centre, Copenhagen, Denmark). The forest map is used to illustrate the relative strength of the effect. The funnel plot is used to illustrate the bias because the number of trials exceeds 10. If a significant difference is detected, a random effects model will be used.

#### 2.5.8 Assessment of reporting biases

We will use a funnel plot to detect report bias. If more than 10 trials are included, the funnel plot will be used to assess the reported bias. If the funnel plot is found to be asymmetrical, analyze the cause using Egger method. We will include all eligible trials regardless of the quality of the method.

#### 2.5.9 Data synthesis

We will use RevMan for all statistical analysis. If considerable heterogeneity is observed, a 95% CI random effects model will be used to analyze the combined effect estimates. Subgroup analysis will be performed with careful consideration of each subgroup if necessary.

#### 2.5.10 Subgroup analysis

There is no presubgroup plan. Subgroup analysis was performed based on control interventions and different outcomes.

#### 2.5.11 Sensitivity analysis

Based on sample size, heterogeneity quality, and statistical models (random or fixed-effect models), we will perform sensitivity analysis.

#### 2.5.12 Grading the quality of evidence

The quality of evidence for all outcomes will be judged by the Grading of Recommendations Assessment, Development, and Evaluation working group approach. Bias risk, consistency, directness, precision, publication bias, are aspects of our assessment. High, medium, low or very low represents the 4 levels of evaluation.

## 3 Discussion

CLBP is one of the most common reasons for physician visits in the United States. Most Americans have experienced CLBP, and approximately 1 quarter of U.S. adults reported having LBP lasting at least 1 day in the past 3 months.^[33]^ CLBP is associated with high costs, including those related to health care and indirect costs from missed work or reduced productivity.^[34]^ A large number of studies have proved that AT is effective, safe, operable, low-cost and promising for CLBP.^[18,19,35]^

The evaluation of this systematic review will be divided into 4 parts: identification, the inclusion of literature, data extraction and comprehensive analysis of data. According to the Cochrane method, this study is based on the analysis of clinical RCT evidence at home and abroad, searching and screening the main electronic literature database with evidence-based medical evidence, providing clinicians with more convincing evidence in decision-making, to better guide clinical treatment.

## Author contributions

**Conceptualization:** Guilong Zhang.

**Methodology:** Xinling Wang.

**Software:** Leixiao Zhang.

**Supervision:** Yanli Deng.

**Validation:** Yang Yu.

**Writing – original draft:** Guilong Zhang, Leixiao Zhang, Yanli Deng, Yuquan Shen.

## References

[1] Tulder MV Becker A Bekkering T Chapter 3. European guidelines for the management of acute nonspecific low back pain in primary care. Eur Spine J 2006;15:S169–91. 16550447 10.1007/s00586-006-1071-2 PMC3454540

[2] Petzke F Welsch P Klose P Opioids in chronic low back pain. A systematic review and meta-analysis of efficacy, tolerability and safety in randomized placebo-controlled studies of at least 4 weeks duration. Schmerz (Berlin, Germany) 2015;29:60–72. 10.1007/s00482-014-1449-8 25503883

[3] Qaseem AWilt TJ McLean RM Noninvasive treatments for acute, subacute, and chronic low back pain: a clinical practice guideline from the American College of Physicians. Ann Internal Med 2017;166:514–30. 28192789 10.7326/M16-2367

[4] Hoy D Bain C Williams G A systematic review of the global prevalence of low back pain. Arthritis Rheum 2012;64:2028–37. 22231424 10.1002/art.34347

[5] GBD 2016 Disease and Injury Incidence and Prevalence Collaborators. Global, regional, and national incidence, prevalence, and years lived with disability for 328 diseases and injuries for 195 countries, 1990–2016: a systematic analysis for the Global Burden of Disease Study 2016. Lancet (London, England) 2017;390:1211–59. 10.1016/S0140-6736(17)32154-2 PMC5605509 28919117

[6] Deyo RA Rainville J Kent DL What can the history and physical examination tell us about low back pain? JAMA 1992;268:760–5. 1386391

[7] Hoogendoorn WE Van Poppel MNM Bongers PM Systematic review of psychosocial factors at work and private life as risk factors for back pain. Spine 2000;25:2114–25. 10954644 10.1097/00007632-200008150-00017

[8] Linton SJ A review of psychological risk factors in back and neck pain. Spine 2000;25:1148–56. 10788861 10.1097/00007632-200005010-00017

[9] Hoy D Brooks P Blyth F The epidemiology of low back pain. Best Pract Res Clin Rheumatol 2010;24:769–81. 21665125 10.1016/j.berh.2010.10.002

[10] Hashemi L Webster BS Clancy EA Length of disability and cost of work-related musculoskeletal disorders of the upper extremity. J Occup Environ Med 1998;40:261–9. 9531097 10.1097/00043764-199803000-00008

[11] Maher C Underwood M Buchbinder R Non-specific low back pain. Lancet (London, England) 2017;389:736–47. 10.1016/S0140-6736(16)30970-9 27745712

[12] Walker BF Muller R Grant WD Low back pain in Australian adults: the economic burden. Asia Pac J Public Health 2003;15:79–87. 15038680 10.1177/101053950301500202

[13] Maetzel A Li L The economic burden of low back pain: a review of studies published between 1996 and 2001. Best Pract Res Clin Rheumatol 2002;16:23–30. 11987929 10.1053/berh.2001.0204

[14] Dagenais S Caro J Haldeman S A systematic review of low back pain cost of illness studies in the United States and internationally. Spine J 2008;8:8–20. 18164449 10.1016/j.spinee.2007.10.005

[15] Maniadakis N Gray A The economic burden of back pain in the UK. Pain 2000;84:95–103. 10601677 10.1016/S0304-3959(99)00187-6

[16] Committee on Advancing Pain Research, Care, and Education, Board on Health Sciences Policy, Institute of Medicine. Relieving pain in America: A blueprint for transforming prevention, care, education, and research. 2011; The National Academies Press, Washington DC. 22553896

[17] Huang LC Auricular Medicine: A Complete Manual of Auricular Diagnosis and Treatment, Auricular International Research and Training. Orlando, Fla, USA: 2005.

[18] Yeh CH Suen L K Shen J Changes in sleep with auricular point acupressure for chronic low back pain. Behav Sleep Med 2016;14:279–94. 26244591 10.1080/15402002.2014.981820

[19] Yeh CH Kwai-Ping Suen L Chien LC Day-to-day changes of auricular point acupressure to manage chronic low back pain: a 29-day randomized controlled study. Pain Med 2015;16:1857–69. 25988270 10.1111/pme.12789

[20] Oleson T Auriculotherapy Manual: Chinese and Western Systems of Ear Acupuncture. 3 edition Kidlington, UK: Churchill Living Stone; 2003.

[21] World Health Organization. WHO Report of the Working Group on Auricular Nomenclature. France, Lyons:1990.

[22] Wu C Liu P Fu H Transcutaneous auricular vagus nerve stimulation in treating major depressive disorder: a systematic review and meta-analysis. Medicine 2018;97:e13845. 30593183 10.1097/MD.0000000000013845 PMC6314717

[23] Yeh CH Chien LC Chiang YC Auricular point acupressure for chronic low back pain: a feasibility study for 1-week treatment. Evid Based Complement Alternat Med 2012;2012:383257. 22811745 10.1155/2012/383257 PMC3395299

[24] Suen LK Wong TK Chung JW Auriculotherapy on low back pain in the elderly. Complement Ther Clin Pract 2007;13:63–9. 17210513 10.1016/j.ctcp.2006.10.005

[25] Oleson T Auriculotherapy Manual: Chinese and Western Systems of Ear Acupuncture. 4th ed. Edinburgh, Scotland: Churchill Livingstone, Elsevier; 2014.

[26] Shamseer L Moher D Clarke M Preferred reporting items for systematic review and meta-analysis protocols (PRISMA-P) 2015: elaboration and explanation. BMJ 2015;350:g7647. 25555855 10.1136/bmj.g7647

[27] Liberati A Altman DG Tetzlaff J The PRISMA statement for reporting systematic reviews and meta-analyses of studies that evaluate health care interventions: explanation and elaboration. J Clin Epidemiol 2009;62:e1–34. 19631507 10.1016/j.jclinepi.2009.06.006

[28] The Cochrane Collaboration, Deeks JJ Higgins JPT Altman DG Cochrane handbook for systematic reviews of interventions version 5.1. 0 (updatedMarch 2011). 2011.

[29] Sator-Katzenschlager SM Scharbert G Kozek-Langenecker SA The short- and long-term benefit in chronic low back pain through adjuvant electrical versus manual auricular acupuncture. Anesth Analg 2004;98:1359–64. 15105215 10.1213/01.ane.0000107941.16173.f7

[30] Zhao HJ Tan JY Wang T Auricular therapy for chronic pain management in adults: a synthesis of evidence. Complement Ther Clin Pract 2015;21:68–78. 25921554 10.1016/j.ctcp.2015.03.006

[31] Ünal Ö Akyol Y Tander B The relationship of illness perceptions with demographic features, pain severity, functional capacity, disability, depression, and quality of life in patients with chronic low back pain. Turk J Phys Med Rehabil 2019;65:301–8. 31893266 10.5606/tftrd.2019.3248 PMC6935732

[32] Alaca N Kaba H Atalay A Associations between the severity of disability level and fear of movement and pain beliefs in patients with chronic low back pain. J Back Musculoskelet Rehabil 2019. 10.3233/BMR-171039 31868657

[33] Deyo RA Mirza SK Martin BI Back pain prevalence and visit rates: estimates from U.S. national surveys, 2002. Spine 2006;31:2724–7. 17077742 10.1097/01.brs.0000244618.06877.cd

[34] Andersson GB Epidemiological features of chronic low-back pain. Lancet (London, England) 1999;354:581–5. 10.1016/S0140-6736(99)01312-4 10470716

[35] Hunter RF McDonough SM Bradbury I Exercise and auricular acupuncture for chronic low-back pain: a feasibility randomized-controlled trial. Clin J Pain 2012;28:259–67. 21753728 10.1097/AJP.0b013e3182274018

